# Design of a randomized controlled trial on the effects of Counseling of mental health problems by Occupational Physicians on return to work: the CO-OP-study

**DOI:** 10.1186/1471-2458-7-183

**Published:** 2007-07-26

**Authors:** David S Rebergen, David J Bruinvels, Allard J van der Beek, Willem van Mechelen

**Affiliations:** 1 EMGO Institute, VU University Medical Centre, Department of Public and Occupational Health, Postbus 7057, 1007 MB, Amsterdam, The Netherlands

## Abstract

**Background:**

Mental health problems often lead to prolonged sick leave. In primary care, the usual approach towards these patients was the advice to take rest and not return to work before all complaints had disappeared. When complaints persist, these patients are often referred to psychologists from primary and specialized secondary care. As an alternative, ways have been sought to activate the Dutch occupational physician (OP) in primary care. Early 2000, the Dutch Association of Occupational Physicians (NVAB) published a guideline concerning the management by OPs of employees with mental health problems. The guideline received positive reactions from employees, employers and Dutch OPs. This manuscript describes the design of a study, which aims to assess the effects of the guideline, compared with usual care.

**Methods/Design:**

In a randomized controlled trial (RCT), subjects in the intervention group were treated according to the guideline. The control group received usual care, with minimal involvement of the OP and easy access to a psychologist. Subjects were recruited from two Dutch police departments. The primary outcomes of the study are return to work and treatment satisfaction by the employee, employer, and OP. A secondary outcome is cost-effectiveness of the intervention, compared with usual care. Furthermore, prognostic measures are taken into account as potential confounders. A process evaluation will be done by means of performance indicators, based on the guideline.

**Discussion:**

In this pragmatic trial, effectiveness instead of efficacy is studied. We will evaluate what is possible in real clinical practice, rather than under ideal circumstances. Many requirements for a high quality trial are being met. Results of this study will contribute to treatment options in occupational health practice for employees on sick leave due to mental health problems. Additionally, they may contribute to new and better-suited guidelines and stepped care. Results will become available during 2007.

**Trial registration:**

Current Controlled Trials ISRCTN34887348

## Background

### Common mental health problems and productivity loss

Common mental health problems can affect functioning to such an extent that they can lead to work absenteeism and presenteeism. These may result in productivity loss [1]. The prevalence of absenteeism due to mental health problems is reported to be between 10 and 18%, which causes extensive societal and financial costs [2-7]. Up to ninety percent of absenteeism is caused by minor, stress-related, mental health problems [3,4,6]. A small, but substantial part (over 20%) of these 'common' mental health problems result in long lasting productivity loss. In the Netherlands associated costs are enormous (9.407 billion Euros in 2004)[8,9].

### Stress management in Dutch occupational health care

For employees with common mental health problems, health care utilization is mainly restricted to primary care. In the Netherlands, primary care is regularly given by general practitioners  and occupational physicians (OPs). The majority of these OPs are working for commercially operating Occupational Health Services (OHSs). Each Dutch employee has to visit their OP for rehabilitation purposes when they are on sick leave. Therefore, the Dutch OP has a perfect opportunity to play a central role in the diagnosis and treatment of employees with common mental health problems. However, OPs often lack time and skills to deal with these employees [10,11]. Consequently, the approach of Dutch OPs towards employees on sick leave due to mental health problems has been minimal. The usual initial advice given by OPs and GPs towards these patients has been to take rest and only return to work when all complaints have disappeared. When complaints persist, these patients are often referred to psychologists from primary and specialized secondary care. Treatment by these psychologists is mostly symptom based rather than focusing on return to work. Most employees are not insured for these treatments. Recent Dutch, and Scandinavian, studies suggest that an inactive primary care that easily refers to specialized secondary care, may cause 'referral' delay in recovery [10-14]. In addition, referrals to specialized secondary care can be expensive for employees and employers, as they have to pay the price. As a consequence, patients may not get the optimal care they need.

### Stress management by occupational physicians

As an alternative to usual care, ways have been sought to activate the Dutch OP in primary care. As the OP is visited by each employee with or at risk of common mental health problems, the OP has a key role to detect them and influence their return to work. Therefore, a renewed position of the OP was introduced in a national evidence based guideline regarding the management by OPs of employees with mental health problems [15,16]. The guideline was published by the Dutch Association of Occupational Physicians (NVAB) in 2000. It promotes an active attitude and activating approach, instead of a minimal role of the OP. The guideline received positive reactions from employees, employers and Dutch OPs. However, there is reason to believe that the actual implementation of the guideline lags behind its acceptance, which questions the effectiveness of the guideline in practice [17,18].

### Study rationale/Objective

The aim of this study is to examine the effectiveness of the care by Dutch OPs according to the new common mental health guideline. The guideline may improve the effectiveness of occupational rehabilitation among workers with common mental health problems. This study will focus on the effects of training in the guideline on the skills of the OP, resulting in positive effects on return to work and treatment satisfaction.

## Methods/Design

### Study design

In a randomized controlled trial (RCT) the effect of the 'Dutch national guideline on the management of employees with mental health problems by OPs' was evaluated. The focus of this study is to examine the new, more active role of Dutch OPs according to the new guideline. Therefore, subjects in the intervention group were treated by OPs, who were trained to provide treatment according to the guideline. The control group received usual care, with minimal involvement of the OP and if applicable, access to treatment by a psychologist.

The first hypothesis of this study is that the intervention will lead to health gain for employees on sick leave due to common mental health problems. This will result in faster recovery, less stagnation and less referrals to psychologists. Counseling, instead of symptom based treatment, will result in earlier return to work and consequently a decrease of productivity loss. The second hypothesis is that the intervention will additionally lead to relatively more treatment satisfaction of the employee, the employer and the OP. The third hypothesis is that a decrease of productivity loss and prevention of expensive referrals to secondary care, will reduce costs.

The recruitment of participants for the study started in January 2002 and ended in January 2005. There was a one year follow-up. The study was funded by the Dutch Ministry of Internal Affairs and Kingdom Relations, and the Insurance Agency on Medical Guidance of the Dutch Police (DGVP). The study design, protocol and procedures were approved by the Medical Ethics Review Committee of the VU University Medical Centre.

### Participants

#### Setting

This study was conducted with the cooperation of the Dutch police force, which is an organization with a relatively high incidence of mental health problems. These problems are mainly work related as police work has inevitable risks and stress may develop as 'part of the job' [19]. The employer of the Dutch police, the Ministry of Internal Affairs, tried to provide an optimal care and was open to alternative effective treatments. Each Dutch police employee was insured by the insurance company, the DGVP. DGVP tried to provide optimal usual care by partly financing referrals by OPs of police employees with mental health problems to a commercial psychotherapeutic centre as part of a protocol. Therefore, these police departments and their occupational health care provided a representative study population.

This intervention was developed for the occupational health care setting with its typical case load of common mental disorders. Two police departments were chosen because they had contracts with the same private OHS, i.e. Commit. Consequently, uniformity in treatment was more secured. Commit is one of the largest OHSs in the country. The police departments, i.e. Zaanstreek-Waterland and Hollands Midden, were located in the South-West of the Netherlands. Hollands Midden comprises approximately 1700 employees; Zaanstreek-Waterland approximately 800, totalling a source population of 2500 police employees.

Because we wanted to prevent employees with chronic disability to participate in the study, each employee on sick leave due to mental health problems before the start of the study in 2002, was detected by the OHS. They received a treatment by a psychologist in secondary care funded by the DGVP.

#### Recruitment and selection of the participants

**Figure 1:**
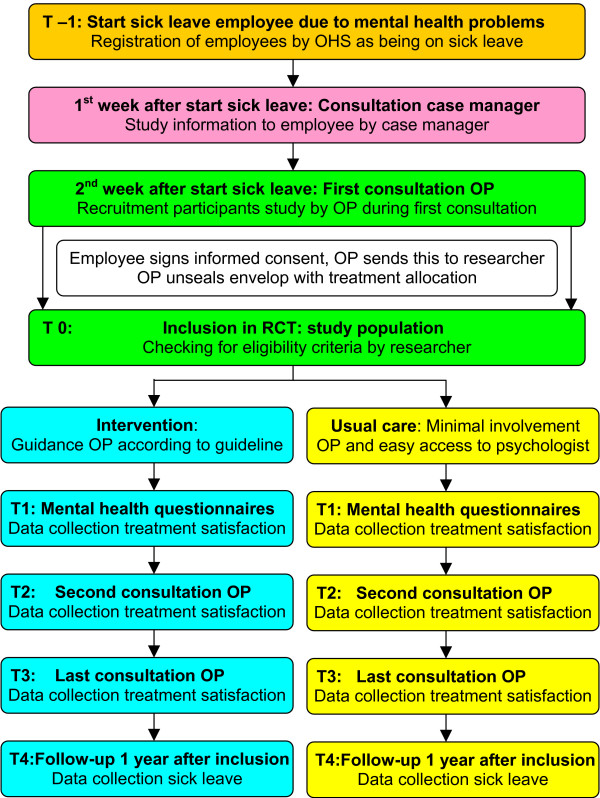
Flow chart of time line, study design and return to work.

Regularly, employees were registered on their first day of sick leave by the OHS (figure 1). Since January 2002, each employee on sick leave due to mental health problems was invited to meet with a case manager of the OHS within one week. This case manager informed the employee about the study and planned a consultation with an OP in the first two weeks of sick leave. To enhance recruitment one of the researchers (DB), who was allowed to check the registration system of the OHSs, informed the OP when a potential participant would come for consultation. Each employee who consulted an OP, and was still on sick leave due to mental health problems, was then asked by the OP to participate in the study. After an employee had signed informed consent during this consultation (T0), the OP unsealed a study envelope containing the allocated treatment for the patient, and sent the signed informed consent to the researcher (DR). In the same consultation the employee received the baseline questionnaires and was asked to return this questionnaire to the researcher after completion.

#### In- and exclusion criteria

As the guideline focuses on all kinds of mental health problems, we aimed to include employees with a broad range of mental health problems consulting their OP. Employees were included if they met the following inclusion criteria:

• Mental health problems according to the diagnosis of the OP

• Sick leave at the moment of inclusion

• Sick leave period did not start before 2002.

Exclusion criteria were the same as stated in the OP-guideline:

• Mental health symptoms that were caused by somatic illness

• Disagreement between OP and employee about the diagnosis

• Lack of confidence in the relation between OP and employee

The application of the exclusion criteria was dependent on the OPs expert judgement. To prevent selection bias, employees were not included of whom the period of sick leave started before 2002.

### Randomisation

Block randomisation (size 50) was done on the patient level before the start of the study using SPSS. The randomisation results were sealed in 250 consecutive envelopes. The OPs were informed about the study procedure and received sealed numbered envelopes, in which the treatment was stated which they had to provide. They were allowed to open an envelope only after an employee voluntarily signed an informed consent. Then the OP told the participant to which treatment her or she was assigned.

To minimize the risk of irregularities by letting OPs open their treatment concealment themselves, randomisation was checked by an independent researcher (AvdB) one year after the start of the study. At the end of the study this procedure was repeated by checking the treatment allocation of all the in- and excluded persons.

### Blinding

Participants, employers and OPs were not blinded for the intervention. The researchers were blinded and did not know the treatment allocation of the employee, to prevent any influence on the study procedure. As this is an effectiveness study researchers were blinded for protocol compliance as well, to make the trial as realistic as possible. Blinding of the gathered sick leave and medical files data was secured, since these measurements were gathered from the automated databases of the police constabularies, the insurance agency and the OHSs.

### Interventions

#### Usual care

In this study the aim was to compare usual care of employees with mental health problems with an intervention. Usual care consisted of minimal involvement of the OP and access to treatment by a psychologist, as this represents daily practice. As the aim was to deliver the best optional care to our study population, optimal usual care was provided in this study. This consisted of the advice to OPs to refer to a psychologist, whose treatment was fully funded by the DGVP. The OP could refer to a psychologist working for a commercial multidisciplinary rehabilitation center, i.e. De Gezonde Zaak (DGZ), as this was part of an agreement with the OHS Commit. A patient was only referred if according to the expert judgement of the OP this made sense to the health condition of this person.

DGZ is one of the largest Dutch commercial psychotherapeutic intervention centers, which focuses on return to work of the employee. DGZ is located in different parts of the country. Besides physical therapists, around 100 psychologists are working for this organization. The psychologists are working according to cognitive behavioral principals. The standard therapy offered was based on protocols of the Dutch Institute for Work and Stress [20].

#### Intervention

The intervention consisted of treatment by OPs according to the guideline of employees on sick leave due to mental health problems. The guideline promotes a more active role of the OP as case and care manager facilitating return to work of the employee. The guideline is based on an activating approach, time contingent process evaluation and cognitive behavioral principles. The latter mainly concern stress inoculation training and graded activity and aim to enhance the problem-solving capacity of patients in relation to their work environment.

The guideline focuses on four aspects of the management of mental health problems. First, an early and activating guidance by the OP is promoted, in which return to work is part of the recovery process, even if the mental health problems are not related to work. Second, a simplified classification of mental health problems is introduced, with only four categories: 1) adjustment disorder (distress, nervous breakdown, burnout), 2) depression, 3) anxiety, and 4) other psychiatric disorders. Third, the OP acts as case manager, who is stimulated to be a care manager by counselling employees with adjustment disorders and work-related problems. Fourth, the OP performs a time contingent process evaluation and intervenes when recovery stagnates.

OPs participating in the study received training in the guideline before the study started. During this training, consisting of a three-day course with 10–15 other OPs, knowledge about and practice in working with the guideline were educated and exchanged [21]. The course reflected the guideline by training OPs in multiple cognitive-behavioral prescriptive interventions, to stimulate the patients' acquisition of problem solving skills, and to structure the patients' daily activities. Information was given and OPs were trained to differentiate between adjustment disorder and depression, anxiety and other psychiatric disorders. Questionnaires were introduced, which can be helpful in making an accurate diagnosis. In addition, a graded activity treatment approach was introduced, which was based on a three stages model. This treatment approach resembles stress inoculation training, a highly effective form of cognitive behavioral treatment [16]. In the first stage, there is emphasis on information: understanding the origin and cause of the loss of control. Patients are also stimulated to do more non-demanding daily activities. In the second stage, patients are asked to draw up an inventory of stressors and to develop problem solving strategies for the causes of stress. In the third stage, patients put these problem solving strategies into practice and extend their activities to include more demanding ones. The patients' own responsibility and active role in the recovery process was emphasized and the same goes for the importance of an early start of the intervention aimed at the acquisition of coping skills and at regaining control. Problem solving activities according to a time contingent scheme were educated and practiced. The OPs were free to choose the specific tools for use in each phase of the process. The training course was given by four persons: an experienced OP/psychologist, a psychologist/therapist, an experienced general practitioner/researcher on emotional distress, and a psychiatrist.

#### Co-interventions

Co-interventions cannot always be avoided. In case of post traumatic stress disorders, patients were also referred to a specialized trauma centre according to a special protocol of the DGVP. As the rehabilitation centre DGZ worked with multiple disciplines, it is possible that some patients in the control group received a combined intervention for mental and physical complaints by a psychologist and a physical therapist. In both the intervention and control groups co-interventions were registered in the medical files of the OHS and the database of the DGVP. These data can be used to adjust for co-interventions in the final multivariate analyses.

#### Compliance

As this is an effectiveness trial, we tried to mimic a realistic situation in both treatment groups. Therefore, no activities were undertaken to improve the actual treatment compliance by the OP with the allocated treatment. Treatment compliance of the OPs was examined by measuring guideline adherence and assessing the proportion of referrals by the OP in both groups to the psychologist of DGZ. Patient compliance of the treatment was examined by registering no shows of patients during consultations with the OP were, as this may give information about the willingness of the patient to adhere to the treatment.

#### Contamination

As randomization was done on patient level, OPs which were trained in the guideline treated all participants. Obviously this situation created a risk of treatment contamination between the groups. The trained OP treated an employee in the intervention group according to the guideline, as far as this happens in practice. The same OP treated an employee in the control group with minimal involvement and if applicable, direct referral to a psychologist. A cross-over learning effect may have happened in the control group, since the OP can adhere to the guideline in this group as well. The other way around, the OP may have referred an employee in the intervention group to a psychologist as well. The guideline promotes this in case of stagnation in recovery or in case of severe mental health problems of the employee. However, we tried to maximize the contrast by creating a situation in which referral to the psychologist in the control group was always granted by the insurance company (DGVP).

### Primary outcomes

#### Return to work

**Figure 2:**
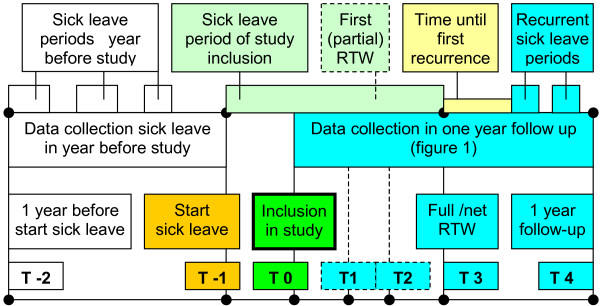
Timeline of measured sick leave data of a potential participant.

Return to work (RTW) was chosen as the primary outcome in this study [22]. A follow-up time of one year after inclusion was chosen, as effects of the intervention on return to work were expected to happen in this period. The RTW-outcomes are visualised in a time line in figure 2.

The primary outcome measure described in our study is full RTW: i.e. duration of sick leave due to mental health problems in calendar days from the first day of sick leave to full return to work in own or equal earnings (table 1). In addition, the net return to work to own or equal work was measured. This is the net duration of sick leave due to mental health problems in hours of full work absenteeism from the first day of sick leave to full return to work with own or equal earnings. The difference with full RTW is that the hours of partial return to work and % contract working hours (36 hours = 100%) are converted into the number of hours of full work absenteeism [23]. This may result in a more precise measure of RTW, when compared to full RTW. As figure 2 shows, net and full RTW consider the time period between T0 and T3, in which data collection by questionnaires took place (figure 1).

**Table 1:** Measured data and their instruments and timing of data collection

**Data**	**Instrument**	**Base-line**	**Follow up**			
**Data related to primary outcomes**		**T0**	**T1**	**T2**	**T3**	**T4**
Return to work						
Full Return to work	Database company	X			X	
Net return to work	Database company	X			X	
First (partial or full) return to work	Database company	X	X	X	X	
Time until 1^st ^recurrence	Database company	X				X
Number and days of recurrences of sick leave	Database company				X	X
Total days of sick leave during one year follow up	Database company	X			X	X
Treatment satisfaction						
Treatment satisfaction of employee	Questionnaire		X	X	X	
Treatment satisfaction of employer	Questionnaire		X	X	X	
Treatment satisfaction of the OP	Questionnaire				X	

**Data related to secondary outcome**						

Cost effectiveness						
Direct costs of treatment						
• Consultations OP and treatment OHS	Medical files OHS	X	X	X	X	
• Consultations of participating psychologist centre	Medical files centre	X	X	X	X	
• Consultation of general practitioner	Insurance company	X	X	X	X	
• Consultations psychiatrist/psychologist/alternative therapist	Insurance company	X	X	X	X	
• Medication	Insurance company	X	X	X	X	
Indirect costs of lost productivity						
• Net lasting RTW and earnings	Database company	X			X	
• Replacement	Database company	X			X	

**Data related to prognostic measures**						

I) Personal characteristics						
Gender, Age	Database company	X				
Severity disorder: DASS/HADS (effect modifier)	Questionnaires		X			
Work-relatedness of the disorder	Medical files OHS	X				
Sick leave in year before inclusion (effect modifier)	Database company	X				
II) Treatment						
Treating occupational physician (OP)	Medical files OHS	X				
Diagnosis made by the OP	Medical files OHS	X				
Guideline adherence of the OP	Medical files OHS	X	X	X	X	
III) Work characteristics						
Type of function	Database company	X				
Number of working hours	Database company	X				
Police constabulary	Database company	X				

Another primary outcome variable that is part of the RTW process is first RTW: i.e. the duration of sick leave due to mental health problems in calendar days to first (partial or full) return to own or equal work. Other variables are related to recurrences of sick leave periods in the one year follow-up (T4)(table 1). These variables are the time in calendar days until the first recurrence of sick leave takes place and the number of and days during recurrences [21]. Total days of sick leave during follow-up is a primary outcome variables as well.

There was double registration of sick leave data, as both the employer and the OHS have their own registration system. The aim was to compare data of both systems, with the sick leave data of the employer as the 'golden standard'. This additional effort was done as reliable sick leave data are hard to get, since there is a known discrepancy with self-reported sick leave [24,25].

#### Treatment satisfaction

Treatment satisfaction is a relevant outcome measure in occupational health care and therefore another primary outcome [26]. Higher patient satisfaction is related to better patient compliance and can improve the quality of OHSs. To date, most researchers assume that patient satisfaction is best defined as a patient's evaluation of aspects of a health care service based on the fulfilment of patient expectations. Since patients, employers and health care providers (OPs) are all involved stakeholders in the RTW process, it is important to measure the treatment satisfaction of all of these stakeholders.

Patient and employer satisfaction were measured using a short version of the Patient Satisfaction with Occupational Health Professionals Questionnaire [26]. This questionnaire was designed specifically for measuring satisfaction with occupational health care. It was designed in previous research on the quality of rehabilitation of cancer survivors [27] and transferred to occupational health care in another study on employees with mental health problems [18]. The 13 items of this questionnaire refer to (a) satisfaction in general (2 items), (b) interpersonal approach (4 items), (c) communication manner (2 items), (d) professional knowledge (5 items), and (e) total satisfaction of the treatment by the OP (13 items). Respondents answered on thirteen statements on a 5-point Likert scale: 'totally disagree – disagree – no opinion – agree – totally agree' (table 2). Because a higher score indicates more treatment satisfaction, item 3,4,9,10 and 12 will be recoded. The patient satisfaction questionnaire was adapted to the situation of the employer, to measure the treatment satisfaction of the supervisor (table 3).

**Table 2:** Treatment satisfaction questionnaire employee

	Items relate to the last consultation the employee has had with the OP*
	**General satisfaction with the OP**
1	I am very satisfied about the contact with the OP
2	In general, contact with the OP made sense
	**Interpersonal approach by the OP**
3	The OP can be more respectful to me
4	The OP is more interested in the employer's, than my point of view
5	The OP seems interested in me as a person
6	The OP treats me in a pleasant manner
	**Communication manner of the OP**
7	The OP is good in explaining his or her opinion about returning to work
8	The OP listens well to what I have to say
	**Professional knowledge by the OP**
9	The OP forces me to return to work, while this is impossible
10	The OP has no experience with my kind of problems
11	The OP gives me good advice about how to deal with my health complaints
12	The OP does not seem professional to me
13	The OP knows what he/she is talking about
1–13	**Total satisfaction of the treatment by the OP **(all 13 items mentioned above)
*	5-point Likert scale from 1 (totally disagree) to 5 (totally agree)
	To increase the readability of this article we have translated the questionnaire from Dutch to English. In the study the Dutch version was used.

**Table 3:** Treatment satisfaction questionnaire supervisor

	Items relate to the last consultation your employee has had with the OP*
	**General satisfaction with the OP**
1	I am very satisfied about the contact with the OP
2	In general, contact with the OP made sense
	**Interpersonal approach by the OP**
3	The OP could be more respectful to me
4	The OP is more interested in the employee's, than the employer's point of view
5	The OP seems interested in me as supervisor
6	The OP treats me in a pleasant manner
	**Communication manner of the OP**
7	The OP is good in explaining his or her opinion about return to work of my employee
8	The OP listens well to what I have to say
	**Professional knowledge by the OP**
9	The OP forces my employee to return to work, while this is impossible
10	The OP has no experience with my kind of problems as being a supervisor
11	The OP gives me good advice about how to deal with the health complaints of my employee
12	The OP does not seem professional to me
13	The OP knows what he/she is talking about
1–13	**Total satisfaction of the treatment by the OP **(all 13 items mentioned above)
*	5-point Likert scale from 1 (totally disagree) to 5 (totally agree)
	To increase the readability of this article we have translated the questionnaire from Dutch to English. In the study the Dutch version was used.

To measure treatment satisfaction of the OPs, OPs filled in an evaluation questionnaire for each employee treated. This questionnaire consisted of 6 items, the first 4 referring to possible barriers in the RTW process and the last 2 items referring to the treatment success of their OHS (table 4).

**Table 4:** Treatment satisfaction questionnaire OP

	**Process evaluation of the treatment by the OP**
1	**Which was the effect of the treatment given by the OHS on the employee,**
a)	related to recovery?*
b)	related to return to work?*
2	**Was the employee cooperative regarding the treatment?**
	a) No, not cooperative; b) Cooperative, but passive; c) Cooperative and active; d) No idea
3	**Was the employer cooperative regarding the treatment?**
	a) No, not cooperative; b) Cooperative, but passive; c) Cooperative and active; d) No idea
4	What was the influence of the following factors on return to work of the employee?*
a)	Degree of physical work load
b)	Degree of mental work load
c)	Degree of physical work ability of the employee
d)	Degree of mental work ability of the employee
e)	Support by supervisor
f)	Support by colleagues
g)	Support by employer
h)	Work motivation of the employee
i)	Job control of the employee
j)	Relationships at work between employee and employer
k)	Duration of curative treatment
l)	Advices of the curative sector
m)	Waiting lists in the curative sector
n)	Inadequate sickness behaviour of the employee
o)	Psychosocial situation of the employee
p)	Financial situation of the employee
q)	Home situation of the employee (including care tasks)
r)	Remaining, not work-related, factors
s)	Practical (including organizational) options to work accommodations
t)	Financial circumstances employer
u)	Other factor, namely...
5	**To what extent are you satisfied by the treatment of the OHS, related to**
a)	Treatment effectiveness?**
b)	Treatment process?**
*	Response range: 1. obstructive ; 2. no effect or influence ; 3. supportive
**	7-point Likert scale from 0 (totally dissatisfied) to 6 (totally satisfied)
	To increase the readability of this article we have translated the questionnaire from Dutch to English. In the study the Dutch version was used.

### Secondary outcome

#### Cost-effectiveness measures

Cost-effectiveness of the intervention is a secondary outcome and was evaluated from the employers and the health care insurance company's perspective (expenditures for the employer and insurance company, respectively), as they are responsible for covering the costs of sick leave and treatment [28]. Direct costs of health care treatment are (table 1): (a) consultations of OPs and other OHS-professionals, (b) consultations of the psychologists from DGZ, (c) consultations of general practitioners, (d) consultations of a psychiatrist and/or psychologist and/or alternative therapist not participating in the study, and (e) medication related to the treatment of mental health problems [29].

Indirect costs are not related to health care, but are costs as a consequence of absence from work because of sickness: sick leave, disability and or death of productive persons. Costs of lost productivity caused by (partial) sick leave due to mental health problems were calculated from the net number of days of sick leave and lost earnings, as provided by the employer. Since our study took place in occupational health care and since most costs were caused by sick leave, extra efforts were made to gather reliable data on sick leave [30].

### Prognostic measures

Prognostic measures and potential confounders were searched for in the literature [16,31,32]. The following prognostic measures were selected and will be taken into account as potential confounders (table 1): I) personal characteristics: (a) gender, (b) age, (c) disorder severity, based on mental health symptoms (DASS, HADS), (d) work relatedness of sick leave on the moment of inclusion, e) total days of sick leave in the year before the inclusion (figure 1); II) treatment characteristics: (f) treating OP, (g) guideline adherence by the OP, (h) referral behaviour of the OP; III) work characteristics: (i) type of function (executive vs. administrative), (j) working hours (part-time vs. full-time), and (k) police department (Zaanstreek-Waterland vs. Hollands Midden).

#### Depression Anxiety Stress Scale (DASS)

To measure mental health complaints at baseline in this study, the Depression Anxiety Stress Scales (DASS) were used [33]. The structure of the DASS seems to support the view that both anxiety disorders and depression need to be distinguished from adjustment disorders in spite of their communality. The psychometric properties of this instrument appear to be sound enough to be applied to both healthy and psychiatric populations. Therefore, the psychometric properties of the DASS are suitable for use in an occupational health care setting. Moreover, convergent and divergent validity have been shown to be satisfactory [34].

The employees participating in this study filled in a self-report questionnaire that comprises the DASS-42, which takes 7 minutes to complete. The DASS-42 consists of 42 symptoms divided into three subscales of 14 items: depression scale, anxiety scale, and stress scale. Participants rated at baseline the extent to which they had experienced each symptom over the previous week on a four point Likert scale ranging from 0 (did not apply to me at all) to 3 (applied to me very much, or most of the time).

Based on the results of their study on employees with mental health problems in occupational health care, Nieuwenhuijsen et al. [34] developed cut-off scores to divide the DASS-rates into four categories: stress, depression, anxiety, and depression/anxiety. The cut-off scores are > 12 on symptoms of depression and > 5 on symptoms of anxiety.

#### Hospital Anxiety Depression Scale (HADS)

The HADS is a 14-item screening scale that measures the presence of anxiety and depressive states [35]. It contains two 7-item subscales: a depression subscale and an anxiety subscale, each item being scored on a four point Likert scale (0–3) that applies to the previous week. The HADS has been developed as a screen for detecting depressive and anxiety disorders in hospitalised patients. Items referring to symptoms that may have a physical cause (for example, weight loss or insomnia) are not included in the scale. Because a higher rate indicates more mental health symptoms, item 1,3,5,6,8,10,11 and 13 will be recoded.

The HADS is easily administered as a self-report measure as it usually takes 3–5 minutes to complete. A total score (out of a possible 21) for each subscale is then calculated. Zigmond et al. [35] recommended cut-off points with scores less than eight on either of the two subscales to be non-cases and scores between eight and ten as borderline cases.

#### Guideline adherence by the OP

The aim of this study is to examine the effectiveness of the management by Dutch OPs, under the expectation that (training in) the guideline will lead to additional skills for the OP and consequently to positive outcomes. To explore our hypothesis that the guideline leads to additional skills and outcomes, we examined the performance by the OP according to the guideline (guideline adherence). Guideline adherence by the OP was checked by means of an audit of the medical files. Guideline adherence was defined as the total score on ten validated performance indicators for the treatment of each participant by the OP (table 5)[11,18,36]. For each performance indicator, we used validated criteria. If a criterion was not met, the case was assigned 1 for that performance indicator. If all applicable criteria for a performance indicator were met, the resulting score was 0 for that case. The medical files of all the participants were assessed on if they met the criteria of the different indicators (0=adequate care; 1=deviant care). In this way an average performance rate was obtained for each performance indicator. Furthermore, a total score of all performance indicators was calculated (guideline adherence). Guideline adherence was dichotomized into adequate adherence and deviant adherence. Adherence was considered deviant if three or more performance indicators were assigned a score of 1, and adequate if less than three performance indicators had a score of 1.

**Table 5:** Performance indicators guideline adherence and their criteria 1 = deviant care, NA = Not applicable

**PI 1**	**Assessment of symptoms**	**Score**
Criteria:	1. Presence or absence of essential symptoms of anxiety disorder and depressive disorder should be noted in file	
	2. Presence or absence of distress symptoms (fatigue, concentration problems, sleeping problems, and emotional reactivity) should be noted in file	
	*One or both criteria not met within 2 consultations?*	PI1 = 1

**PI 2**	**Correct diagnosis**	

Criteria:	1. Diagnosis should be noted in file	
	2. Diagnosis should be correct:	
	- IF adjustment disorder: at least one psychological distress symptom should be noted in file	
	- IF depressive disorder: at least one essential symptom AND five depressive symptoms should be noted in file	
	- IF anxiety disorder: at least one anxiety disorder should be noted in file	
	3. Diagnosis should not be missed if criteria above apply	
	*One or more criteria not met within 2 consultations?*	PI2 = 1

**PI 3**	**Evaluation curative care**	

Criteria:	1. Treatment in the curative sector, or its absence, should be noted in file	
	2. IF patient receives treatment, THEN the OP should evaluate whether this treatment is effective	
	*One or both criteria not met within 2 consultations?*	PI3 = 1

**PI 4**	**Assessment work-related causes**	

Criteria:	1. the work-related causes, or their absence, should be stated in file	
	*One or both criteria not met within 2 consultations?*	PI4 = 1

**PI 5**	**Evaluation of work disabilities**	

Criteria:	1. Functional limitations in home or work environment, or their absence, should be stated in file.	
	2. Work activities of patient should be noted by OP	
	3. OP should assess whether patient is limited in his work functioning	
	4. IF patient has work limitations, THEN OP should assess other impediments for return to work (such as problems in home situation or with supervisor)	
	One or both criteria not met within 2 consultations?	PI5 = 1

**PI6**	**Interventions targeted at individual**	

	1. Intervention aimed at the individual should be noted or be referred - IF adjustment disorder, THEN OP should start interventions OR should refer patient to psychologist/social worker/general practitioner OR should consult with practitioner giving current treatment - IF anxiety disorder OR depression OR other psychiatric disorder, THEN OP should refer patient to psychologist/social worker/general practitioner OR should consult with practitioner giving current treatment	
	*Criterion not met within 3 consultations?*	PI6 = 1

**PI7**	**Interventions targeted at organisation**	

	IF work is a causal, eliciting or maintaining factor in the mental health problem, THEN OP should intervene in the work organisation (confer with supervisor/personnel officer)	
	*Criterion not met within 3 consultations?*	PI7 = 1
	*IF work is neither causal, eliciting nor maintaining factor in mental health problem*	PI7 = NA

**PI8**	**Interventions targeted at providers of care in curative sector**	

	1. IF treatment in curative sector is lacking and deemed necessary, THEN OP should start interventions targeted at the individual OR refer patient to psychologist/social worker/general practitioner	
	2. IF treatment in curative sector is not effective, THEN OP should consult with practitioner giving current treatment	
	*One or both criteria not met within 3 consultations?*	PI8 = 1
	*IF patient receives effective treatment in curative sector*	PI8 = NA

**PI9**	**Advice on return to work**	

	1. Advice on return to work should be provided by OP	
	2. IF no impediments for return to work are present, THEN OP should advise full or partial return to work	
	*One or both criteria not met at each consultation?*	PI9 = 1
	*Patient already (partially) returned to work?*	PI9 = NA

**PI10**	**Timing of consultations**	

	1. First consultation should be within 3 weeks from first day of sickness absence	
	2. IF patient has not yet completely recovered, THEN next consultation should be within 4 weeks from previous consultation	
	*Criterion 1 not met at first consultation OR criterion 2 not met at consultation 2 or 3?*	PI10 = 1

Additionally, this audit gave us information about treatment compliance by the OP (guideline adherence) in both groups. In this way contamination between the study groups was studied as well. Adherence in the intervention group was considered compliant if there was adequate adherence. Guideline adherence in the control group was considered compliant if there was deviant adherence.

The performance indicators will be assessed on their criteria by three independent researchers, resulting in a dichotomised score on guideline adherence for each employee (adequate versus deviant).

### Data collection

The participants had to complete the mental health questionnaires (DASS, HADS) on the moment (T1) after they signed the informed consent (T0) (figure 1) (table 1). At the same time a questionnaire had to be filled in about their satisfaction with the treatment of the OP (T1). This questionnaire was sent again to the participant by the researcher (DR) after their second consultation with the OP (T2) and after the last consultation with the OP, at the moment of full RTW (T3). If T2 and T3 happened at the same moment, T2 was considered as T3. The questionnaires were returned to the researcher after completion in pre-stamped envelopes. The same was done for the supervisors of the participants, who received a questionnaire about their treatment satisfaction at the same moments (T1, T2 and T3). The OP received for each participant after the moment of full RTW another questionnaire to assess their treatment satisfaction (T3).

Baseline characteristics of the participants such as gender, age, marital status, work characteristics (type of function, hours working, part/full time), sick leave data and costs of work incapacity of the participants were gathered from records of the police constabularies, the latter after one year follow-up. Data about direct costs of treatment and medication of the employee were obtained after one year follow up from the insurance company of the police, the DGVP.

Guideline adherence and the according performance were based on data of the medical files of the participant, gathered from the databases of the participating OHSs. The data of the medical files were made anonymous and were transferred to an Access database, to select the relevant data of the medical files.

### Study population: sample size and power analysis

In order to detect a relevant difference in survival analysis on our primary outcome return to work, nQuery Advisor [37] was used to calculate the sample size. Proportions used to determine the sample size needed, were analysed from sick leave data of the police constabularies in 1999. In 1999, 286 employees were registered as being on sick leave due to mental health problems, which was 6.6 % of the total sick leave registrations. Their duration of sick leave in 1999 was 35.5 % of the total volume of sick leave, with an average of three months per case. With a power of 90%, at a 0.05 level, a two-sided log-rank test for equality of survival curves was done, assuming a difference between the intervention and control group proportion still on sick leave after one year of 0.25. This test indicated that a sample size was needed of 107 in each group. Assuming a dropout rate of 20%, inclusion of a total of 268 patients was necessary to statistically detect a clinically relevant difference.

### Data analysis

All analyses will be conducted according to the intention-to-treat principle and will be performed on the patient level. To examine the success of randomization, descriptive statistics will be used to compare the baseline measurements of the two groups. If necessary, analyses will be adjusted for prognostic dissimilarities.

The evaluations on the effectiveness of the guideline compared to usual care will be performed with two tailed tests at a significance level of 5% (P < 0.05). To examine differences in the data on RTW, we will use Kaplan Meier's and the Cox proportional hazard regression for recurrent events. The general idea behind this analysis is that the different time periods are analysed separately adjusted for the fact that the time periods within one patient are dependent. Recurrences of sick leave for any reason during follow-up will be added to the Cox proportional hazards model with the time to event approach, in which only the transitions from no treatment success (sick leave) to treatment success (full RTW) are taken into account [38].  In this model, the state of sick leave until the moment of inclusion will be added as a covariate. Number and days of sick leave periods in the year before inclusion will be added as a potential effect-modifier.

In a linear regression model treatment satisfaction during treatment (T2) and after treatment by the OP (T3) will be measured as respectively a short term and a long term effect and differences will be compared between the groups. Treatment satisfaction at the start of the treatment (T1) will be added as a covariate, even as the treatment group to examine differences in effects between the groups. The levels of treatment satisfaction of both the employees and their supervisors will be compared for the different moments with a chi-square test. Pair-wise correlations will be used to compare treatment satisfaction of the employee and their supervisor on the different moments.

In the Cox and linear regression models potential treatment differences by the OPs and their OHSs will be taken into account by means of nested dummy variables. The police constabulary the employee works for, will be put into the model as a binomial variable. Differences in sick leave patterns in the year before inclusion and in the severity of the mental health problems (DASS/HADS-scores) will be put into the model as potential effect modifiers.

To assess whether protocol deviations will cause bias, the results of the intention-to-treat analyses will be compared to per-protocol analyses. A process evaluation will be done, based on the assessment of guideline adherence by means of performance indicators [18]. For each performance indicator potential effects on our primary outcomes will be measured.

Indirect costs can be calculated using the friction cost approach (friction period 122 days) and the human capital approach, based on income as provided by the employer or as derived from function, age and gender [30]. Bootstrapping will be used for pair wise comparison of the mean groups to calculate mean differences and confidence intervals in costs and cost-effectiveness ratios for all interventions. All these analyses will be conducted in SPSS 14.0, Excel and, if necessary, in Strata.

## Discussion

### Methodological considerations

#### External validity of study results

The study population, Dutch police employees, has a higher risk of getting into stressful situations than regular workers [19]. This is caused by a relatively high workload and emotional pressure, and to a certain extent this reflects that police employees have other occupational risks than the regular worker population.

Additionally, the Dutch police workforce underwent two big reorganizations in the last ten years. This resulted in some negative consequences as problems with the internal communication, especially between employee and employer [39]. Because of the Volendam fire in January 2001, the police department Zaanstreek-Waterland had been exposed to extra professional risks on mental health problems and traumas one year before the start of the study [40]. As the study population will not be fully representative of the general working population, external validity of study results may be limited and caution has to be taken in generalizing the results.

Unfortunately, we cannot rule out selection bias. This may have occurred as the OPs were asked to select employees to participate in the study. Because the treatment of the OP depends on the randomization, an OP could have been tempted to forfeit the randomization procedure. As mentioned earlier, a check was made to detect this possible selection bias, and eventual irregularities would or will be noticed. Also, selection bias may have been introduced because participants completed the mental health symptoms questionnaires (DASS and HADS) after the consultation of inclusion. The consultation with their OP might have changed their point of view on their mental health problems and consequently, their response behaviour.

Still, these disadvantages regarding the ability to generalize our study results do not outweigh the advantages of this study population. This project was developed for the occupational health care setting with its typical case load of stress-related mental disorders, and not for specialized care, in which patients have more clearly defined mental disorders. The police is an organization with a relatively high incidence of common mental health problems and is therefore an interesting and representative target population. In addition, the police is a homogenous population that was treated in a confident manner, has a uniform sick leave registration, is connected to one insurance company, and has a well-defined 'usual care'.

#### Contamination between study groups

In this pragmatic study design we examine the effectiveness of occupational health care by OPs who are trained in a practice guideline, compared to usual care. The best situation would be to randomize on physician level and patient level. Due to the limited number of participating OPs this was not possible, hence randomization was done on patient level. Consequently, there was a risk of treatment contamination between the groups. However, contrast between the treatment groups was maximized by free access to the psychologist in usual care as this was granted by the insurance company (DGVP). Treatment adherence will be examined by per-protocol analyses of guideline adherence by the OP. Furthermore, the current method allows us to consider the pragmatic effectiveness and to avoid much interference with daily practice of consulting hours.

#### Evaluation of productivity loss

In our cost-effectiveness evaluation we will not consider productivity loss due to sick leave prior and after the episode of sick leave due to mental health problems as proposed by Brouwer et al. [41]. Considering productivity loss prior and after the episode of sick leave can lead to an increase in estimated production losses of about 16%. In this study productivity loss during the RTW process has been measured.

Productivity loss is not only influenced by the cause of sick leave, but also by the type of work. Some jobs can only be performed in case of full functioning. Police employees, for instance, are called off sick leave only when they can perform all necessary tasks. In all other cases they are still on sick leave, for instance if they have a restriction in work on the street or wearing a gun. Since the start of our study in 2002 better methods in calculating costs have become available [30]. The availability of instruments for the measurement of productivity losses in recent years will give a better estimate of costs in this study.

### Prospect on outcomes

In this trial effectiveness instead of efficacy is studied. We will evaluate what is possible in real clinical practice, rather than under ideal circumstances. As a consequence, mental health state may have varied between the participants. Through subgroup analysis on severity of complaints and levels of distress, measured by the DASS and HADS, we will classify possible high or low risk groups for prolonged sick leave within this heterogeneous group. Identification of a high-risk group for non-recovery may lead to better suited guidelines on stepped care and treatment.

Finally, many requirements for a high quality trial are being met. Results of this study will contribute to treatment options in occupational health practice, for employees on sick leave due to mental health problems. In addition, they may contribute to new and better-suited guidelines and stepped care. Results will become available during 2007.

## Abbreviations

DASS = Depression Anxiety Stress Scale

DGVP = Insurance Agency on Medical Guidance of the Dutch Police

DGZ = De Gezonde Zaak; Dutch commercial multidisciplinary rehabilitation centre

HADS = Hospital Anxiety Depression Scale

NVAB = Dutch Professional Organization of Occupational Physicians

OHS = Occupational Health Service

OP = Occupational Physician

RCT = Randomized Controlled Trial

RTW = Return to work

## Competing interests

DB worked for the participating OHS as OP until September 2005, besides being a researcher.

## Authors' contributions

DB developed the study design and acquired the funding of the study. WvM and AvdB advised on the content of the design and co-ordination of the trial, and in writing the article. DR and DB conducted the study, DR wrote the article. All authors provided comments on the drafts and have read and approved the final version.

## Pre-publication history

The pre-publication history for this paper can be accessed here:


